# Optimal Management of a Pregnant Patient with Rheumatic Heart Disease

**Published:** 2018-08-30

**Authors:** J. Brendan Mitchelson, Douglas E. Cleveland

**Affiliations:** University of Kansas School of Medicine-Wichita, Department of Anesthesiology

**Keywords:** rheumatic heart disease, obstetric anesthesia, mitral stenosis, case report

## INTRODUCTION

Rheumatic heart disease remains the number one worldwide cause of maternal cardiac complications in pregnancy.^1^ Since symptoms of rheumatic fever typically do not present until the fourth or fifth decade, the pathophysiologic changes associated with pregnancy may cause as many as 25% of these women to first experience symptoms during pregnancy. For this reason, it is important that obstetric anesthesiologists remain aware of the disease, its complications, and management of valvular lesions throughout the birthing process. The normal physiologic changes of pregnancy cause unique problems to the mother with underlying cardiac disease.^2^ Intravascular volume and cardiac output (CO) increase while systemic vascular resistance (SVR) decreases to preserve normal mean arterial pressure (MAP). During labor, each uterine contraction results in an auto transfusion of blood, resulting in even higher CO._3_ Likewise, pain and apprehension can lead to sympathetically mediated increases in SVR, heart rate (HR), and CO causing further stress.^2^ Yet, the greatest stress comes immediately after delivery when uterine contraction and involution can increase CO by as much as 80% above third trimester values.^3^

With all these changes, one must realize how valvular disease is affected during pregnancy. In general, regurgitant lesions are tolerated better due to the increase in intravascular volume and the decrease in SVR, thus improving forward flow of blood through the valves.^2^,^3^ In contrast, stenotic lesions are tolerated poorly due to the inability to increase CO through a stenotic valve in the setting of increased intravascular volume and increased preload.^2^

In patients with rheumatic heart disease, mitral stenosis is the most common heart lesion.^1^ When these patients become pregnant, the hypervolemia and increased HR can increase the transmitral pressure gradient, leading to increased left atrial volume and pressure. Pressure can be transmitted to the pulmonary vasculature, resulting in pulmonary edema and in severe cases pulmonary hypertension, a significant risk during pregnancy as it can cause right heart failure.^4^ Further, the chronically dilated left atrium has a propensity to disrupt the cardiac conducting system and cause supraventricular tachycardia, 1 a detrimental event in patients with mitral stenosis who rely on the atrial kick to augment preload. Overall, these factors often cause the previously undiagnosed and asymptomatic patient to develop symptoms during pregnancy, and, in severe cases, experience profound cardiac decompensation.

## CASE REPORT

A 30-year-old Hispanic female, Gravida 11, Para 3-1-6-4, at 33 weeks pregnant presented with a complaint of “increasing pressure in her abdomen”. The patient was not in labor, but her history included a “leaky heart valve” which was described as rheumatic fever. She previously had been told not to have further pregnancies. She had no history of anesthesia or epidural, and no medication other than a prenatal vitamin. The patient lived in a small town about one hour outside of the city. Her chart noted poor medical compliance. She denied chest pain, shortness of breath, presyncope, or palpitations. On exam, a diastolic murmur was appreciated, most notably at the apex. There was no jugular venous distension or edema, and her lungs were clear on auscultation.

An echocardiogram showed moderate mitral stenosis with severe mitral regurgitation, severe pulmonary hypertension, moderate aortic insufficiency with no evidence of stenosis, and chronic diastolic heart failure with an ejection fraction of 60%, clinically well compensated at the time. The patient returned home with plans for close surveillance and for postpartum transesophageal echocardiography.

Two weeks later the patient returned at 35 weeks pregnant with the same abdominal pressure complaints and again was found not to be in labor. As she had missed a follow-up appointment, she was admitted and watched as an inpatient until delivery due to noncompliance with her checkups and to avoid delivery with her heart disease at a small hospital an hour away. She was placed on venous thromboembolism prophylaxis with subcutaneous heparin 10,000 units BID. The cardiology and obstetric teams rounded on her daily. Early in her stay, the patient complained of palpitations and was placed on continuous telemetry, which showed occasional premature atrial and ventricular contractions. These improved without medication.

Nine days after admission, a multidisciplinary joint meeting was held between obstetrics, cardiology, and anesthesia to discuss a plan for delivery. Cardiology noted that the patient appeared well compensated and optimized for delivery. The most worrisome heart lesion was the stenotic mitral valve, and the best management was to keep the patient euvolemic, preferring slight hypovolemia to hypervolemia. She would be scheduled for induction of labor a couple weeks later in her 39th week of pregnancy. The team agreed an epidural block would be placed and titrated slowly for pain management. She would be encouraged to avoid Valsalva maneuvers during delivery with plans for forceps-assisted vaginal delivery to shorten the second stage of labor. Tentatively, a bilateral tubal ligation was scheduled for after delivery. Post-operatively, cardiology would begin loop diuretics to ensure diuresis and minimize the risk of hypervolemic complications after uterine involution.

On the day of induction at 08:44, an epidural catheter was placed in the usual manner with an initial negative test dose. The epidural was set with a rate of 10 mL/hr of 0.125% bupivacaine with 2 μg/ml fentanyl. The patient experienced minimal drop in blood pressure after epidural placement to a nadir of 95/50 mmHg, while HR remained about 90 beats per minute (bpm). As she remained stable, bupivacaine was bolused manually in increasing doses throughout the morning and the sensory level block gradually crept to T7 bilaterally by 11:00 and T6 bilaterally by 12:30 when the cervix reached full dilation. Toward the end of delivery, the patient began to experience chest pain. Her vital signs remained relatively unchanged throughout.

Stage two of labor was expedited with forceps as planned, and a healthy baby girl was delivered at 12:56. The placenta was delivered four minutes later. A 12-lead electrocardiogram obtained at this time, showed normal sinus rhythm at 91 bpm with bi-atrial enlargement and left ventricular hypertrophy, essentially unchanged from admission. The patient received oxytocin and misoprostol, and blood loss was noted to be 350 mL. After delivery, the patient’s chest pain gradually resided. The epidural catheter was left in place with basal rate continued for possible tubal ligation surgery later that day.

As the chest pain had resolved and the patient felt better, postpartum tubal ligation was initiated. When the patient arrived in the operating room at 14:25, her BP was 110/40 mmHg with HR of 90 bpm. When the obstetrician arrived, the patient had continued to bleed postpartum, losing approximately 740 mL more blood. She had not been resuscitated due to the obstetrician’s concern of the complications of hypervolemia, namely heart failure. Therefore, she was resuscitated with lactated ringers (LR) and given two 100 μg doses of phenylephrine. The patient responded well, BP increased appropriately, and the epidural catheter was bolused at 14:45 with 10 mL of 2% lidocaine over 15 minutes. Surgery started at 14:55. The patient required a 20 mg and a 30 mg dose of esmolol when HR exceeded 100 beats per minute, but otherwise tolerated the procedure well. A total of 900 mL of LR was given throughout. The patient remained stable in the recovery unit and the epidural catheter was removed. The anesthesiologist called the cardiologist to give a report, and diuretics were started that night as planned. The patient diuresed well, her chest pain never returned, and on postpartum day three cardiology declared her stable for discharge and she left the hospital. After discharge, the patient was referred to a cardiac surgeon for possible mitral valve surgery.

## DISCUSSION

This case showed the importance of communication between the multiple specialties required to care for complex patients. The multidisciplinary meeting before delivery allowed concerns to be addressed and a plan to be made, as well as prepare to manage complications. When communication breaks down, for instance being unaware of the further blood loss causing hypotension, critical factors affecting treatment may be left out. But when effective communication takes place, such as timely hand off with the cardiologist after surgery, the patient can be started more efficiently on diuretics to optimize her fluid status. Clearly, with good communication, patient care is improved.

In general, when caring for parturients with valvular lesions, a cesarean delivery should be reserved for obstetric indications only,^1^,^2^ as it is associated with more blood loss, increased risk of wound infection, post-operative immobility, and thrombosis.^1^ Euvolemia should be maintained with strict monitoring of intake and output. Lumbar epidural anesthesia is preferred to control pain and limit hemodynamic changes due to sympathetic tone, and to limit the urge to push.^1^ Local anesthetics should be titrated slowly, as a sudden decrease in preload or SVR may be tolerated poorly when the patient develops reflex tachycardia.^2^ As in our patient, the addition of opioids to the local anesthetic mixture may improve pain control without adding to sympathetic blockade, thus worsening SVR. Likewise, an adequate block can decrease patient Valsalva maneuvers, which cause the undesirable effect of increased SVR and may cause circulatory overload. Assisting delivery with forceps or vacuum extraction also minimizes the need for Valsalva maneuvers.^1^

Due to the fact that arrhythmia could cause significant decompensation, continuous telemetry is required.^1^ Anticoagulation is utilized to prevent systemic embolism. Along with the added hypercoagulability of pregnancy, patients with a dilated left atrium and a lesion such as mitral stenosis, where supraventricular tachycardia is not uncommon, are at significant risk of left atrial thrombus formation and stroke. In our patient, unfractionated heparin usually is utilized after 36 weeks gestation, or two to three weeks before expected delivery, due to its shorter half-life and the ability to rapidly reverse. Heparin can be safely discontinued four to six hours before delivery. Similarly, this is another reason to prefer vaginal delivery, as improved mobility postpartum decreases thromboembolism risk.

With regards to mitral stenosis, perhaps the most important point is to maintain sinus rhythm if present pre-operatively and to prevent tachycardia.^3^ Time required for adequate left ventricular diastolic filling is prolonged, thus it is more reliant on sufficient diastolic time along with atrial kick.^2^ For the most part, our patient maintained normal heart rate with adequate pain control throughout, and esmolol was utilized when appropriate to control tachycardia. Ephedrine should be avoided as it may result in tachycardia. The pressor of choice is phenylephrine, as it has little effect on uteroplacental perfusion and the reflex decrease in HR it is known to cause can be beneficial in patients with mitral stenosis.

In our patient, who was well compensated and asymptomatic before labor, increased vigilance, but not necessarily invasive monitoring, was required.^2^ If she had been symptomatic prior to labor, the stress of delivery and increase in postpartum blood volume could have put her at serious risk of cardiovascular collapse, and an arterial line may have been necessary. Serial echocardiography also may be beneficial in this setting. As mentioned, one of the most stressful moments on the cardiovascular system is immediately after delivery when uterine involution greatly increases blood volume and cardiac output. Similarly, as the sympathetic block wears off, the intravascular load may worsen and be tolerated poorly by patients with stenotic lesions and fixed cardiac output. In our patient, diuresis shortly after surgery was important, as many maternal complications can happen in the week after delivery, or even months later.^5^

One thing to consider in this case was whether the timing of tubal ligation was appropriate. In patients with mitral stenosis, a moment of great strain on the heart shortly after delivery may increase intravascular volume and overload the cardiovascular system leading to pulmonary edema. Our patient already was known to have pulmonary hypertension. In addition, even though the electrocardiogram was unchanged, our patient developed chest pain during delivery. On the other hand, she had a history of poor medical compliance, and if she were to become pregnant again, a delivery would be even riskier. After discussing contraceptive options with the obstetric team, the patient decided on permanent sterility. As the epidural catheter was in place and the patient did not appear volume overloaded, it was determined she could undergo surgery safely. When the patient was hypotensive, it was appropriate to delay until after fluid resuscitation had improved her blood pressure and shown her to be fluid responsive. This stabilized her prior to another epidural bolus and provided further evidence that her hypotension was not due to volume overload and heart failure.
